# A transdisciplinary account of water research

**DOI:** 10.1002/wat2.1132

**Published:** 2016-01-14

**Authors:** Tobias Krueger, Carly Maynard, Gemma Carr, Antje Bruns, Eva Nora Mueller, Stuart Lane

**Affiliations:** ^1^ Integrative Research Institute on Transformations of Human‐Environment Systems (IRI THESys)Humboldt‐Universität zu BerlinBerlinGermany; ^2^ School of GeoSciencesUniversity of EdinburghEdinburghUK; ^3^ Centre for Water Resource SystemsVienna University of TechnologyViennaAustria; ^4^ Governance and Sustainability LabTrier UniversityTrierGermany; ^5^ Institute of Earth and Environmental ScienceUniversity of PotsdamPotsdamGermany; ^6^ Institute of Earth Surface DynamicsUniversity of LausanneLausanneSwitzerland

## Abstract

Water research is introduced from the combined perspectives of natural and social science and cases of citizen and stakeholder coproduction of knowledge. Using the overarching notion of transdisciplinarity, we examine how interdisciplinary and participatory water research has taken place and could be developed further. It becomes apparent that water knowledge is produced widely within society, across certified disciplinary experts and noncertified expert stakeholders and citizens. However, understanding and management interventions may remain partial, or even conflicting, as much research across and between traditional disciplines has failed to integrate disciplinary paradigms due to philosophical, methodological, and communication barriers. We argue for more agonistic relationships that challenge both certified and noncertified knowledge productively. These should include examination of how water research itself embeds and is embedded in social context and performs political work. While case studies of the cultural and political economy of water knowledge exist, we need more empirical evidence on how exactly culture, politics, and economics have shaped this knowledge and how and at what junctures this could have turned out differently. We may thus channel the coproductionist critique productively to bring perspectives, alternative knowledges, and implications into water politics where they were not previously considered; in an attempt to counter potential lock‐in to particular water policies and technologies that may be inequitable, unsustainable, or unacceptable. While engaging explicitly with politics, transdisciplinary water research should remain attentive to closing down moments in the research process, such as framings, path‐dependencies, vested interests, researchers’ positionalities, power, and scale. *WIREs Water* 2016, 3:369–389. doi: 10.1002/wat2.1132

For further resources related to this article, please visit the WIREs website.

## INTRODUCTION

Water is a basic human requirement and a matter of sheer survival, it is a natural raw material and an economic commodity, it is a matter of course and a lifestyle product, it is an instrument of power and a scarce good—too much or too little of it may lead to disaster. (Anne Dombrowski, THESys Water Dialogue)

Water is at the heart of many of today's global challenges. There is a gross disparity in people's access to water and sanitation that is not just a matter of biophysical availability, but also technological, economic, and political possibility. Domestic demands compete with ecology, agriculture, and other industries for water resources that are ultimately finite. These competitions play out via economic and political mechanisms on scales from the local to the global. Droughts as well as floods threaten livelihoods; and overexploitation, overregulation, and pollution of water bodies exacerbate the problems further. Hydraulic infrastructure, such as large dams, built to secure water or energy supply, has social and ecological implications. These challenges attest that water is at the same time a material, political, economic, and cultural entity (what political ecologists of water call the hybrid nature of water).^1^ Water can entail different meanings with sharply material consequences. As a recent special issue on water ontologies puts it,^2^ ‘water can be and become a border, a resource for regeneration, a foundation for empire, a means of nation building, and a material linkage between past and present’ (Ref 2, p. 485). In this study, we focus on the different ways of knowing water, asking how these come to be through the interplay of material, political, economic, and cultural factors and how they may be brought together in water research.

An interdisciplinary approach to water research has long been considered indispensable for understanding the multifaceted issues surrounding water. It is no coincidence that water management agencies employ biologists, ecologists, economists, geologists, historians, lawyers, planners, political scientists, and sociologists as well as engineers and hydrologists.^3^ However, water knowledge exists beyond that of scientists and other certified experts, which may demand some form of participation of stakeholders and citizens in water research and management.^4^, ^5^ Such knowledge may both unsettle the existing wisdom of those disciplines concerned with water; but may also itself need to be unsettled so as to bring into sharp focus the material nature of water (i.e., water as more than just discourse), or its manifestation in ways (e.g., space or time scales) not necessarily appreciated by stakeholders or citizens (e.g., the impacts of future climate change).

The term transdisciplinarity, although not typically associated with water research, usefully generalizes and extends the terms interdisciplinarity and participation for describing the practices of water research that interest us here (see Box 1). For this study, we adopt Pohl's approach B to transdisciplinarity as a starting point,^6^ which (1) focuses on socially relevant issues; (2) integrates disciplinary paradigms; and in which (3) critical participatory research becomes the defining feature for progression from interdisciplinarity. As such, socially relevant issues, instead of disciplines, define the frame of enquiry; and actors from different scientific disciplines (interdisciplinarity) and from civil society and the private and public sectors (participation) collaborate in the production of water knowledge. We will explore in this paper how transdisciplinarity has been taking place in water research and how it could be developed further.

BOX 1 **TRANSDISCIPLINARITY**

Concepts of transdisciplinarity have been developed in many research traditions, without a single precise definition.^68^, ^75^ Klein^76^ identified three dominant discourses of transdisciplinarity: transcendence (transdisciplinarity as an epistemological project toward unity of knowledge); problem solving (transdisciplinarity as a method of knowledge production); and transgression (transdisciplinarity as a form of critique and reimagination of modes of knowledge and its discourses and institutions). Pohl^6^ offered a compelling unifying typology, in which the semantic progression from multi‐, over inter‐ to transdisciplinarity is shared by all definitions, while they differ in what exactly constitutes this progress. *Multidisciplinarity* in this typology is the joint working of different scientific disciplines on an issue, virtually in parallel, without any philosophical and methodological integration. *Interdisciplinarity*, in turn, aims at the integration of disciplinary paradigms. We suggest in this paper, following Barry and Born,^17^ that interdisciplinary research may be more productive when it goes beyond mere synthesis and takes on agonistic forms that challenge disciplinary foundations. For Pohl, *transdisciplinarity* in its least developed form (type A) is a form of ‘interdisciplinarity plus’ which not only integrates disciplinary paradigms but does so as defined by socially relevant issues. Type B adds participatory research as a third key feature. Type C has the search for unity of knowledge as the third feature. Transdisciplinary research traditions draw from a variety of concepts that parallel those found in water research. The motivation for transdisciplinary water research are commonly socially relevant issues where facts are uncertain, values in dispute, stakes high, and decisions urgent,^77^, ^78^ so‐called ‘wicked problems.’^79^, ^80^ In response to these types of problems, participatory methods^47^, ^81^ have been developed to foster coproduction of knowledge^6^, ^82^ and social learning^68^, ^83^ among different actors.

The study is organized as follows: the first section (*From Integrative to Agonistic Interdisciplinary Water Research*) gives an overview of the philosophical, methodological, and communication challenges of integration in interdisciplinary water research and argues for a more agonistic relationship between philosophical positions. The second section (*How Water Science Reflects (and Reproduces) Cultural, Political, and Economic Context*) reviews Science and Technology Studies (STS) of water that have analyzed how water science is influenced by and influences social order (what STS scholars call coproduction). Because our focus is the knowledge production process, the scope of this review is limited to detailed accounts of the actual knowledge resulting from coproduction processes and how this could have turned out differently. How this knowledge then loops back to perform political work is only highlighted by what we consider key papers where necessary for our argument. We do not attempt a comprehensive review of the political ecology of water. In the third section (*When Scientists and Nonscientists Coproduce Water Knowledge Explicitly*), we apply the coproductionist insights and political charge to argue for the explicit coproduction of water knowledge by scientists and nonscientists. In doing so, we give an overview of participatory water research, again limiting ourselves to the knowledge production process. We only bring in lessons from overarching processes of participatory water governance that are relevant for our discussion. A comprehensive review of participatory water governance is given elsewhere. 7 In the fourth section (*The Tension between Opening Up and Closing Down Moments in Transdisciplinary Research*), we reflect on the practices of transdisciplinary water research that have emerged from the literature and our own experience, before setting out a transdisciplinary water research agenda in the conclusions.

We try to cover a broad range of water research literature from political science, STS, hydrology, geography, and anthropology, including research on water governance, hydrological modeling, river restoration, watershed services, water consumption, socio‐hydrology and social–ecological systems, eco‐hydrology, and climate change adaptation. Inevitably, our choice of literature is influenced by our collective physical and social science background in catchment science, hydrological modeling, and water governance. Throughout the article, and especially in *The Tension between Opening Up and Closing Down Moments in Transdisciplinary Research* section, we mix literature insights with reflections on our experiences in a number of transdisciplinary water research projects (Table 1). As the Working Group ‘Transdisciplinarity’ of the scientific decade 2013–2022 of the International Association of Hydrological Sciences (IAHS) on change in hydrology and society, ‘Panta Rhei,’^8^ we consider transdisciplinary perspectives for a balanced conceptualization and study of human–water relations in this and other scientific initiatives.

**Table 1:** List of Transdisciplinary Projects from which Experience is Drawn in the Text

ID	Type of Research	Types of Nonscientists Involved	Who Initiated the Process	Funding	Locality	Time Period	Author Contribution
P1	Ryedale Flood Research Group	Local people for whom flooding was a ‘matter of concern’ recruited through newspapers etc.; since 2009 incorporated into a Defra Best Practice Project	Academics	RCUK Rural Economy and Land Use (RELU) program	Ryedale, North Yorkshire, UK	2007–ongoing	SL
P2	Participatory modeling: nitrogen and phosphorus water quality model	Local government, regional government, public authority, environmental regulator, environmental public body, conservation groups, tourism industry, water company, drainage board, land owners/managers, residents (23)	Academics	RCUK Rural Economy and Land Use (RELU) program	River Thurne catchment, Norfolk, UK	2008–2010	TK
P3	Uckfield Flood Research Group	Local people for whom flooding was a ‘matter of concern’ recruited through newspapers and so on	Academics	RCUK Rural Economy and Land Use (RELU) program	River Uck catchment, Sussex, UK	2008–2012	SL
P4	Catchment management: ecological river flood and drought management	Local government, environmental regulator, conservation group, water company, land owners/managers	Civil Society Organisation	EU Interreg IVB Project	River Eden catchment, Cumbria, UK	2008–2013	SL
P5	Participatory modeling: nitrogen and phosphorus water quality model	Local government, regional government, environmental regulator, environmental public body, conservation groups, tourism industry, businesses, water company, land owners/managers, farm advisors, residents (35)	Academics	RCUK Rural Economy and Land Use (RELU) program	River Tamar catchment, Devon/Cornwall, UK	2009–2011	TK
P6	Participatory modeling: impacts of channel and weir restoration	Local government, conservation group, residents, other local interest representatives	Academics	ESRC/NERC interdisciplinary doctoral scholarship	River Derwent catchment, County Durham/Northumberland, UK	2010–2013	CM
P7	Participatory scenario development based on water quality model; part of a Defra Strategic Evidence and Partnership Project	Environmental regulator, environmental public body, conservation groups, tourism industry, water company, land owners/managers, residents (20)	Civil Society Organisation (The Rivers Trust)	Defra, The Rivers Trust	River Rea catchment, Shropshire, UK	2011	TK
P8	Participatory modeling: farm economic calculator to evaluate land use options	Public authority, environmental regulator, environmental public body, drainage board, land owners/managers (14)	Public authority (Broads Authority)	NERC Knowledge Exchange Fellowship, Broads Authority	Brograve drainage level, Norfolk, UK	2011–2012	TK
P9	Participatory modeling: extension of water quality model by sediment and fecal coliforms; part of the Tamar Pilot of the UK Catchment‐Based Approach to water management	Local government, environmental regulator, conservation group, water company, land owners/managers, residents (9)	Civil Society Organisation (Westcountry Rivers Trust)	NERC Knowledge Exchange Fellowship, Westcountry Rivers Trust	River Tamar catchment, Devon/Cornwall, UK	2012–2013	TK
P10	EU Cost Action ‘Connecting European Connectivity Research,’ Working Group ‘Connectivity & Society’	Local, regional, national and EU administrations for water and land management, farmers, catchment managers	Academics	EU Cost	Across Europe	2013–2017	ENM

## FROM INTEGRATIVE TO AGONISTIC INTERDISCIPLINARY WATER RESEARCH

Water research often takes place across and between traditional disciplines. This is clearly apparent from the numerous research fields that bridge the disciplinary boundaries of, e.g., hydrology, geology, geomorphology, biogeochemistry, ecology, political science, and economy. These disciplines are linked because the systems they each study are coupled. Understanding how changes in one system affect another is essential for reconstructing past societies and environments and for designing future interventions.^9^ However, even disciplines that are philosophically cognate have methodological differences that may hinder interdisciplinary research. For instance, compare the inductive versus deductive traditions of surface water hydrology and groundwater hydrology (also called hydrogeology or geohydrology) where differences begin at the epistemological level, here stemming from the disciplines’ historical foundations in civil engineering and geology, respectively.^10^ In this section, we problematize interdisciplinary integration, focussing in particular on the philosophical, methodological, and communication challenges that have dominated the literature.

Interdisciplinary research has long aimed at the integration of disciplinary paradigms, in which certain philosophical and methodological aspects from a field of study are combined with those from other fields (Box 1), often motivated by innovation or a need to solve a particular problem. While trying to produce an output which is greater than the sum of the disciplinary parts, interdisciplinary efforts have often failed in fully integrating the disciplinary components and have resulted in what may be called multi‐ rather than interdisciplinary research.^11^, ^12^, ^13^ Multidisciplinary studies as understood here consider a range of perspectives, but continue to work with standard disciplinary framings (Box 1). As a consequence, knowledge and management interventions may remain partial or even conflicting, such as when options to mitigate different types of aquatic and atmospheric pollution are appraised in isolation.^14^ Classic examples are social preference studies that feed into physical water resource assessments (e.g., in the form of scenarios), and social acceptance studies that come ‘after the facts.’^15^ In these constellations, social research takes on a subordinate or service role, which is a typical mode of putatively interdisciplinary research.^16^, ^17^ The problem framing is then conceded to an *a priori* natural science foundation, leaving both the social context as well as the consequences of this particular scientific framing unexamined. Another example is the attempt to synthesize physical and social research of human–water relations in general systems theory models (Box 2), which may produce new forms of closure as formerly rich, heterogeneous accounts of humans and water are simplified into a supposedly universal language.^17^ From an anthropological perspective, systems theory ‘is not suited, for example, to expressing the complex and intangible realities of power relations, belief systems, values, understandings of environmental processes, affective responses to place, identity, social relations and so forth’ (Ref 16, p. 14), which can be the most important factors in water management.^16^

BOX 2 **THE HYDROSOCIAL CYCLE AND SOCIO‐HYDROLOGY**

The concept of the hydrosocial cycle draws attention to the internal and external relations between water and society.^1^ Hydrologists have recently also turned to analyzing the *external* water‐society feedbacks within what is called ‘*socio‐hydrology*,’ promoting quantitative methods traditional to hydrology and system dynamics.^84^ In this way, socio‐hydrology is an extension of eco‐hydrology which already adopted system dynamics language from theoretical ecology; and it parallels developments in coupled social–ecological systems modeling more broadly.^85^ Socio‐hydrology aims to observe, understand, and predict future trajectories of the coevolution of coupled human–water systems. In doing so, it reproduces the separation of the hydrological and the social as if they were somehow preconfigured entities. The *hydrosocial cycle* perspective, in contrast, studies how water and society are also related *internally* through the diverse and ever changing meanings, discourses, ideas, and representations attached to water. This includes the particular ways by which hydrological and social knowledge are produced that both reflect and reinforce the social order of which they are part. Coming from a political ecology tradition, hydrosocial cycle scholars have problematized the differentiations of power, knowledge, and water access within society. While the hydrosocial cycle engenders sensitivity to nonscientific meanings and knowledges of water, it does not push a transdisciplinary research program. Neither does socio‐hydrology in its current scope.

In keeping with contemporary scholarship on interdisciplinarity, we find it helpful to locate the primary barrier to interdisciplinary integration along philosophical (ontological and epistemological) divides, rather than reproducing an empirically problematic natural–social science dichotomy. Philosophical divides exist among those studying physical and those studying social processes alike, often hindering researchers studying the same object to collaborate. Conversely, there are cases where few philosophical differences existed between physical and social scientists, and they found it easier to integrate their work.^18^ Connelly and Anderson^18^ and Sharp et al.^12^ located the primary philosophical divide between positivism and postpositivism. For Sharp et al., a positivist approach to domestic water demand is devoted to measuring physical and social phenomena and so can support policymakers through prediction. Underlying rules of the world or of human behavior are taken for granted. The researcher, assumed to stand separate from the research object, seeks to uncover these rules, usually quantitatively. A postpositivist approach, for the authors, places more emphasis on interpreting the particularities that have led to past events, often through a focus upon individual case studies. There is a tendency to reject the idea of general laws, theories or models, focussing instead on everyday practices and how people give meaning to what is happening around them and to what they do. This leads to hermeneutic approaches in which the positionality of the researcher is considered part and parcel of method and interpretation. Broadly speaking, we can thus distinguish between generalizing versus interpretative approaches to water research, and these labels may be more specific than positivist and postpositivist. Between these polar opposites, research traditions differ in more nuanced ways about the degree to which they believe one can abstract from concrete observer situations to rules. In order to circumvent philosophical differences in interdisciplinary projects, the pragmatic suggestion of Connelly and Anderson is for researchers with contrasting philosophical standpoints to explore and develop common criteria for assessing the quality of their work.

A methodological consequence of philosophical differences is that the data collected by some might not be recognized to be useful by others.^12^ Focussing on qualitative versus quantitative data to study water use practices, Browne et al.^19^ suggested that there is a ‘significant gap’ between the largely qualitative studies that are currently used to characterize water consumption trends, and ‘the kinds of quantitative evidence thought to be required for large‐scale strategic planning or policy formation’ (Ref 19, p. 28). In response, the authors advocated methodological pragmatism. Recognizing that a quantitative methodology runs the danger of suggesting generality, which is inconsistent with the interpretative approach of practice theory, they argued that quantitative results could be used purely descriptively. Nonetheless, the authors emphasized the need to complement quantitative with qualitative data to capture the nuances of water use practices and how people give meaning to them, as a purely quantitative approach would be overly partial and reliant on reported behavior rather than an observation of the actual performance of the practices.

The third issue we want to touch upon here is that of communication, which underlies many interdisciplinary research efforts.^11^, ^16^ Different disciplines, and even those taking different approaches within the same discipline (such as modelers and experimentalists), may have different starting points for thinking about specific words, such as ‘water system’ or ‘connectivity.’ For Linton,^20^ the concept of the water system presents an advancement over the classic hydrological cycle in that it includes the human dimension, yet it presents humanity as an overly abstracted, undifferentiated whole. The hydrological buzzword connectivity, in turn, keeps scientists across the environmental disciplines occupied in defining it as a concept, a measure, a phenomenon, a driver, a process, a property, or an extent to which elements of an environmental system are connected in space and time.^21^, ^22^, ^23^ Contradictory meanings prevail in neighboring disciplines such as hydrology, landscape planning, and ecology, which has been identified as the key problem of integrated research on connectivity between those disciplines,^24^ and may result in confused sampling designs of field studies or the incoherent setting up of modeling approaches in joint projects. From our experience in project P10 (Table 1), social scientists and people outside academia appear to link the term connectivity much more to the connectedness of the disciplines themselves, rather than of physical or social entities in space and time.

It is particularly problematic when scientists communicate without noticing that a particular word or terminology has a specific disciplinary interpretation not known or different to scientists from other disciplines. Bracken and Oughton^11^ discussed this problem in terms of disciplinary dialects and different uses of metaphors, which they argued require proper articulation if a shared language is to be developed. Articulation is very much a joint effort of all involved, requiring us, first of all, to say when we do not understand a term or concept. Baveye,^13^ commenting on interdisciplinary collaboration between biologists and hydrologists, noted some familiarization with the literature of the other discipline as a prerequisite for shared language, arguing that there is often insufficient time for this level of engagement. Bracken and Oughton highlighted the forming of trust and respect between researchers, which is seen as important for managing the politics and emotions that come with repeatedly challenging and being challenged in one's disciplinary foundations.

There are more barriers to interdisciplinary integration we did not discuss here, particularly methodological ones such as scale.^11^ But the above examples should suffice to illustrate the philosophical, methodological, and communication barriers that exist. These examples certainly make us wonder whether output‐oriented integration is a realizable and useful aim of interdisciplinary research. Perhaps, it would be more productive to harness the agonistic potential of interdisciplinary processes. Here we refer to what Barry and Born^17^ called the agonistic‐antagonistic mode of interdisciplinary research, which ‘springs from a self‐conscious dialogue with, criticism of or opposition to the limits of established disciplines, or the status of academic research or instrumental knowledge production in general,’ aiming to ‘contest or transcend the given epistemological and/or ontological assumptions of specific historical disciplines’ (Ref 17, p. 12). While this mode of enquiry may be initially motivated by opposition (antagonistic), the term agonistic here emphasizes mutual respect and the eventually productive aspects of this confrontation.

Indeed, the responses to the challenges of interdisciplinarity offered by the researchers we cite above highlight how researchers can be challenged positively in their positions by others, through the process of reflecting upon and learning through their own as well as their joint practices. Sharp et al., using the somewhat weaker term ‘interrelating interdisciplinarity,’ described how qualitative research on water use challenged the underlying assumptions of a demand forecasting model in an interdisciplinary project. The pragmatic call by Connelly and Anderson for common research quality criteria may remain unsatisfactory if the process of elaborating these criteria does not tackle fundamental philosophical differences. Bracken and Oughton described the articulation of terminology very much as an agonistic process. Our own collective experience in interdisciplinary projects, departments, and education programs suggests that a willingness to participate in a discursive culture and a certain terminological precision are requirements for channeling disciplinary antagonism positively. Establishing this culture requires time that is not readily invested by everyone. It also requires continuity in the form of staff or institutional memory that is difficult to maintain when short research contracts are the norm. But once the ground is laid for respectful contestation of epistemological and ontological foundations, agonistic processes may expose the social context and content of any knowledge (*discussed next in the How Water Science Reflects (and Reproduces) Cultural, Political, and Economic Context* section) and thus pave the way for transdisciplinary knowledge practices (*discussed in the When Scientists and Nonscientists Coproduce Water Knowledge Explicitly* section).

## HOW WATER SCIENCE REFLECTS (AND REPRODUCES) CULTURAL, POLITICAL, AND ECONOMIC CONTEXT

Among the most radical social science critiques of natural science philosophy and practice has been the insight that scientific knowledge is not as ‘objective’ as some natural scientists might claim. It is not uncovering a ‘truth’ that is out there waiting to be exposed. Instead, STS have shown that scientific knowledge, like any type of knowledge, embeds and is embedded in a particular social context (what STS scholars call the coproduction of science and social order).^25^ Scientific knowledge is a product of a particular history. Lane^26^ demonstrated this historical contingency for the hydrological concept of Manning's *n*. Krueger et al.^5^ showed for environmental modeling how the modeler makes judgements that are only insufficiently constrained by the material world they model. For the same river basin, e.g., this has meant that different modelers presented with the same dataset have produced very different models and results, according to their personal research culture, experience, and priorities.^27^ While the radical nature of these insights has worn off, they still flag the tension between the freedom of those who decide the means of knowledge production and the constraints and opportunities associated with the social and material context within which that knowledge is produced. What scientific knowledge is produced depends upon how this tension is played out. The important questions to ask are who or what has influenced the production of that particular piece of knowledge, and what this knowledge does in the world, with or for whom. Much as these questions are intertwined, as the term coproduction seeks to imply, in this article on knowledge production we focus on the first set of questions, only bringing in key material on the second set of questions as necessary for our argument. Specifically, we limit our review to detailed accounts of the actual knowledge resulting from coproduction processes, and those that give a sense of how this knowledge could have turned out differently.

While in the examples of Lane and Krueger et al. the social contexts in which the chosen hydrological concepts and environmental models had been produced arose largely from within the scientific community, this process is open to wider cultural, political, and economic influences. It is obvious that the politics of research programs and associated funding streams determines in part the science that academics do. Scientists, both academic and in industry and government, have become more and more business‐like when they market their research in response to funding opportunities. Particular kinds of expertise may thereby circulate more successfully than others just because they meet the market demands more efficiently as well as creating communities of practice that sustain that expertise.^28^ However, the social influences on scientific knowledge production run much deeper. Social context determines how water science is practiced, such as the freedom that a scientist has to innovate,^29^ but also the social constraints that bound their scope of enquiry (e.g., in terms of policy‐directed science vs science‐informed policy).^30^ This is why the concept of the hydrosocial cycle is valuable (Box 2), even if the social influences on water research are only now becoming documented.

A good starting point is the work by Linton on the different conceptions of water, which showed *inter alia* that hydrology as we know it today, and the hydrological cycle as its key analytical frame, arose historically out of the science–politics of 1930s America.^20^ Linton argued that the exclusion of any social dimensions in the circulation of water thereby served to establish hydrology as a pure natural science, presumably following the imperative of the times. Linton identified ‘an internal coherence between this way of knowing and representing water, the consolidation of hydrological expertise, the identification of water as a ‘resource’ to be ‘managed,’ and the power of the state in managing and controlling this resource’ (Ref 20, p. 113). These relationships shall now be explored in more detail with the help of contemporary case studies.

Bouleau^31^ traced the histories of two very different assemblages of hydrological knowledge and corresponding water management paradigms in the Seine and Rhône river basins during the periods 1989–2000 and 1979–1992, respectively. Both stories begin with a political change that presented an opportunity for some scientists and not for others to develop their specific area of research. Personal attachment to the object of study by those that chose to become involved also played a role. The resulting framings of the situations focussed on particular issues of the rivers, point‐source water pollution together with end‐of‐pipe Ammonia reduction in the Seine vis‐à‐vis floodplain morphology and habitats together with large instream flow requirements in the Rhône, and led to the further exclusion of alternative scientific approaches. In both cases, the science that was done avoided subjects that appeared too controversial; e.g., the effect of nuclear power plants on water temperature in the Rhône. The use of preexisting categories and processes, in turn, played in favor of certain political agendas; e.g., the focus on Ammonia in the Seine strengthened the role of water managers which could treat the Ammonia problem end‐of‐pipe while Nitrate related issues, diffuse pollution, catchment scale solutions and those for which these were a concern became marginalized.

When research programs and policies legitimize each other this reinforces both the research methods and the policy guidelines particular to the network of actors involved.^32^ During this process, hydrological knowledge in the form of data, statistics, and models may be molded so as to fit powerful interests, as demonstrated by Fernandez^33^ for the summer low flows of the river Garonne. In other cases, methods may be used inappropriately leading to demonstrably inaccurate results, as shown by Forsyth^34^ for the use of the Universal Soil Loss Equation in northern Thailand. Budds^35^ showed for the case of the La Ligua river basin how a physical water scarcity framing of the responsible government agency led to the commissioning of a purely physical modeling study. Not only did the subsequent policy ignore any limitations of the model, further calculations were added *post hoc* to satisfy the majority of water demands under political–economic pressure. This process reinforced the physical scarcity framing, though contested, and maintained the government agency's position of control over water use. At the same time, the existing unequal pattern of water use that gave rise to the situation in the first place was reproduced. Lane et al.^30^ reported a similar situation for catchment scale flood risk assessment. In their case study, the dominant narrative was that climate change rather than land use management would be the dominant flood risk driver to the 2050s. Closer inspection of the modeling methodology showed that this analysis assumed that development control would effectively constrain floodplain development, i.e., land use. This means the narrative was not simply a neutral one, but one that would need to effect change within the drainage basin so as to reproduce the assumptions behind the model and such that the model could be deemed correct.

Alatout, in a series of articles, traced the historical coproduction of water science with imperial and nation–state borders in historic Palestine. He showed that water became the main object of geological investigation when groundwater resources became politically relevant in postimperial nation–state building. The imperial legacy and the political process of 1930s border drawing thereby structured what could be imagined as the geohydrology that was subsequently researched and, in turn, used to legitimize those borders. The borders were such that surface water was not politically relevant, and it did not feature in these first water resources assessments. Only when it did become relevant in the 1940s vision of an expanded nation–state was surface water, in the form of large‐scale transfer projects, considered in the water resources assessments.^36^ This science was closely linked with the narrative of water abundance that was politically important for nation–state building at the time. Alatout went on to show how in the first decade of the Israeli state the view of abundance gave way to the now familiar water scarcity narrative, and corresponding science, which was enabled by, and legitimized, a turn to centralized water management that was important for nation–state consolidation.^37^ The political work of the physical water scarcity narrative is well documented in other places, too.^35^, ^38^, ^39^

Alatout emphasized partiality of knowledge and persistent uncertainty as the preconditions for overrepresentation and exclusion in scientific accounts. Ignorance, for that matter—that is what we could know but do not—is as much subject to coproduction as what we know. Milman and Ray^40^ demonstrated for the transboundary Santa Cruz aquifer that maintaining a situation of hydrogeological uncertainty and not sharing data on cross‐border groundwater fluxes served the political agendas on both sides that sought to preserve their respective water management narratives. Each party could continue to interpret the sparse data in such a way as to construct a conceptual hydrogeological model that fitted their preexisting perspectives of the situation.

The influence of economic imperatives on science is perhaps most striking in the case of the ecosystem services concept. Robertson^41^ argued that what is a service is fundamentally determined by what can be marketed and sold, which drives the need to measure this service scientifically, not the other way round. Measuring ecosystem services, in turn, has been fraught with uncertainties.^42^, ^43^ Robertson quoted a U.S. National Research Council document from 2005, which asks ecological models explicitly to produce the output that economic models require as input. A quote from an EU‐sponsored report from 2008 asks for scientific simplicity so as to fit with the understanding and implementation ability of ecosystem services retailers. Often, this economic influence is much less explicit, but exists despite continued reluctance by some ecologists and hydrologists to put a measure of value on what many consider invaluable. To be sure, the ecosystem services concept performs a great variety of functions for many actors in different contexts, enables new alliances and brings about discourses and interventions that reinforce certain agendas while marginalizing others.^44^ The ecosystem services discourse is economically and politically useful enough for these actors, including some ecologists and hydrologists, to purport a scientific ability to measure ecosystem services which sooner or later might just become an unquestioned ‘fact.’^43^

## WHEN SCIENTISTS AND NONSCIENTISTS COPRODUCE WATER KNOWLEDGE EXPLICITLY

Our review in the preceding section has sought to illustrate that any prevalent water knowledge could have turned out differently had other cultural, political and economic factors dominated in its production; i.e., if knowledge had been produced in different ways. The political ecologists we cited in particular have insisted that this prevalent knowledge is always contested (overtly or not) by alternative framings, and that people are implicated by this knowledge despite not having had a say in its production. While these insights have often remained descriptive, a form of critique of the traditional portrayal of scientific knowledge, recent scholarship at the intersection of STS and political ecology has begun to encourage scientists to be more reflexive about their own practices and lead them to reimagine the way research is performed.^25^, ^26^, ^45^, ^46^ Following this literature, the coproductionist insights for us hold an argument for transdisciplinary research in which scientists and nonscientists *coproduce* water knowledge explicitly; if there is a democratic deficit in knowledge production then we should open up knowledge production explicitly to those different perspectives, alternative knowledges, and implications that exist. It is this opening up that forces scientists to turn away from their normal networks of knowledge production, i.e., their disciplines, and to build new networks around very different questions and kinds of collaboration.^29^ There is a considerable literature on the so‐called participation of stakeholders, citizens, or the public in water research and governance. For the most part, this literature has not been motivated by the coproductionist critique, apart from a few notable cases.^4^, ^47^, ^48^, ^49^

Participation in water research and governance is typically motivated normatively (people have a right to influence matters that affect them), substantively (bringing diverse perspectives and knowledges together leads to better evidence and policies) or instrumentally (participation leads to greater acceptance of policies and outcomes).^47^, ^50^ Although water research and governance are intertwined, the large body of literature on participation in water governance has been less concerned with participation in knowledge production. Because transdisciplinarity is centered on knowledge production, we focus on the considerably smaller literature on participatory water research here. An overview of participatory water governance is given elsewhere.^7^ For transdisciplinary research, the substantive rationale for participation is of particular relevance because it is concerned with creating a more accurate and comprehensive understanding of the issues being explored. But also the normative rationale may resonate strongly in arguments for bringing problems and interventions to the debate that were not previously considered in order to identify more ethically sound and equitable strategies and counter potential lock‐in to particular water policies and technologies that may be inequitable, unsustainable, or unacceptable.^51^, ^52^, ^53^ This is particularly relevant in the context of sustainable development where multiple objectives exist and water problems are typically contested. For example, problems may not be recognized (such as groundwater pollution), denied to exist by some stakeholders (such as flood risk, water scarcity, or nitrogen pollution) or may only exist for a specific stakeholder (such as microbial pollution or biodiversity reduction). A transdisciplinary approach can help make explicit the varied interests and bring together the scattered and fragmented knowledge held by many different people.^54^

The question that follows is how knowledge coproduction can best be done. Critical for ‘good’ participation is that it strives for a fair and competent process.^55^ Both criteria shall be discussed in turn. ‘Fairness’ here means that anyone who considers themselves potentially affected by the results of the participatory process has equal opportunity to participate, and that every participant has equal opportunity to assert, challenge and influence the final determination of what is considered valid by the group.^55^ In practice, this is an idealistic view. It provides no clear guidance for dealing with existing distributions of power within a society that may impede certain people from contributing.^7^ Nor is it sensitive to the distribution of benefits and concerns that those involved in participation may bring to the process. Participatory research is well recognized as being vulnerable to manipulation by powerful interest groups.^56^, ^57^ Long‐term sustainability may be of lower priority to participants than short‐term financial gain thereby jeopardizing interests with no voice, such as future generations.^58^, ^59^ As is discussed below, this means that questions of representation are critical in evaluating who is involved and who else should be.

‘Competence,’ while resting on minimal standards for cognitive and lingual competence of the participants, emphasizes clear and consistent rules and procedures that promote the competent construction of understanding as part of the participatory process. This includes adequate access to stored knowledge and experiences unfamiliar to the participants, agreed‐upon procedures for translating between knowledge domains and reliable techniques for deciding between conflicting validity claims.^55^ In practice, again, there may be barriers to the immersion of participants in very technical domains such as hydrological modeling (Box 3), which we experienced in project P9 (Table 1). It is naïve to assume that competences, as well as the ability to articulate them, will be evenly distributed among participants at the outset of a transdisciplinary process. This is why it is necessary to reflect upon an ‘entry point’ to the participation. This might be generated through the use of objects,^47^ brought by participants, which demonstrate their personal connection to the issue at stake (Box 4). Such an entry point should be seen as a partial ‘leveling device’ that, unlike conventional approaches to generating scientific knowledge work, privileges the emotive and sensory dimensions of the issue at hand at the expense of the more formal existing knowledge (e.g., reports and scientific contributions) that will tend to reinforce existing framings.

BOX 3 **PARTICIPATORY MODELING**

Participatory modeling is a well‐established mode of transdisciplinary water research, which variously draws on the instrumental, substantive, and normative rationales of participation reviewed in the main text. Hence, participatory modeling efforts need to be carefully interrogated to understand exactly what kind of coproduction is implied. Jonsson et al.^86^ identified six dimensions of participation in the modeling process:

1. What is to be modeled?
2. Who is to be involved in the modeling process?
3. How is the system to be modeled (e.g., through an existing model or through coconstruction of a new model)?
4. How is the model to be set up?
5. How is the model to be used?
6. Where is the participation situated in terms of the wider modeling process?

Review of the literature shows that participatory modeling in water research has actually become an umbrella term for any kind of modeling where there is some kind of engagement between the modeler and what are generally termed ‘stakeholders,’ the latter variously taken as those with professional and those with personal interests in the process. Some key points emerge. First, there are considerably fewer accounts of participatory modeling where the process began, literally, with a blank piece of paper, with little idea as to what was going to be modeled (Jonsson et al., point 1). Examples include Videira et al.^87^ in relation to river basin management, Lane et al.^4^ in relation to modeling options for flood risk reduction, and Giupponi et al.^88^ in relation to flood vulnerability. Even in cases where the problem to be modeled was coframed by the participants, *how* it was going to be modeled was often a decision of the professional modeler.^5^ In this sense, many participatory modeling approaches have been relatively conservative.

Second, given the former observation, it is not surprising that much participatory modeling involves the adoption of existing models (Jonsson et al., point 3), brought in by academics involved in the process, rather than the construction of new models.^89^, ^90^, ^91^ Indeed, the ideas brought into the process as to what is to be modeled may well correlate with what a researcher knows *can* be modeled. In addition, model building is a highly technical activity where there may be a considerable differentiation in the ability of participants to take part. But the role of technical specialists, such as modelers, in participatory processes should be a delegated role that is renegotiable by all participants.^5^, ^69^ There exist technical possibilities for making the model building process more transparent through, e.g., graph–theoretical representations,^91^, ^92^ and even highly technical skills such as computer programming are nowadays widely distributed within society.

Third, accounts of participatory modeling rarely think through who should be involved in the process (Jonsson et al., point 2). This is perhaps where there is a gap between the participatory modeling community and the wider debates surrounding participation discussed in the main text. There is often a clear division of labor (Jonsson et al., point 6) reminiscent of Barry and Born's^17^ subordinate service mode of interdisciplinarity. For instance, Giupponi et al.^88^ described how flood vulnerability modeling tasks were divided between local stakeholders who identified the dimensions of vulnerability and academics who focused on quantifying them into vulnerability indices. In parts, this division of labor is due to the aforementioned technical challenges that limit participation in some elements of the modeling process. Yet, it needs to be challenged if participatory modeling is to engender a deeper understanding of the modeling process, its advantages and limitations among the participants.

Fourth, there is widespread acceptance that participatory modeling helps in the setting up of models (Jonsson et al., point 4) and their validation and use in simulation (Jonsson et al., point 5). Such acceptance reflects one of the critical challenges of hydrological models; their strength is realized through the combination of a supposed generality with the boundary conditions that make them work in particular places. Participatory modeling bridges these two very different kinds of knowledge, providing the boundary information that can be so hard to acquire, and thus making modeling a more effective exercise.^93^, ^94^ For example, Arheimer et al.^89^ showed that inclusion of more detailed local information resulted in very different estimates of nutrient leaching from agricultural land into water courses. Thus, participatory modeling has the potential to bridge two kinds of knowledge systems that easily find themselves in conflict.^95^

In many ways, such use of models reflects a utilitarian view, valuing the role of participation in modeling as a means of producing better models. But there is also evidence of a less utilitarian view, where participatory modeling serves the heuristic purpose^61^ of moving attention away from mere deliberation toward practice and the production of knowledge.^95^ Here, the interest in modeling is less what the model predictions tell us and more the role of the modeling process itself in social learning.^96^ Nevertheless, it is important to retain a critical perspective on participatory modeling. For instance, while participatory modeling may help in making progress in situations of conflict,^97^ there may remain a wider political and cultural economy in the modeling process, such as informal assumptions encoded in models,^5^ which is not clarified when only model predictions are visualized and discussed. To date, there have been surprisingly few critical interrogations of participatory modeling in water research and most of these accounts have been written by those who advocated and instigated the process in the first place.

BOX 4 **ENVIRONMENTAL COMPETENCY GROUPS AND FLOOD RISK MANAGEMENT**

Lane et al.^4^ described the use of Environmental Competency Groups (ECGs) in two river basins (projects P1 and P3, Table 1) as a form of transdisciplinary flood risk management, based upon a series of key principles. First, the focus was upon locations that were controversial; i.e., where the normal procedures of flood risk management had broken down, and where the conflict about what should be done provided a motivating force for engaging people in the process of working out new solutions. Second, local people were recruited for whom flooding was a matter of personal (and not professional) concern, who worked together with academic participants to produce new knowledge in relation to flooding in each locality. Local people were not identified so as to be ‘representative’ as it was recognized that to do so would *a priori* define what was to be represented. Thus, ECGs are not designed to be democratically accountable in some kind of majoritarian sense. Rather, they are designed to construct a new understanding of a problem that may act to slow down or even change the direction of existing thinking, within democratically accountable processes.

Third, the ECGs were not designed to be deliberative. Rather, they aimed to produce new framings of the problem through the production of new knowledge; i.e., they were inherently ‘scientific.’ To do so required an ‘entry point,’ which was the presentation by each group member of an object that showed their personal attachment to flooding. This was critical in showing the distribution of expertise among group members, notably diluting perceptions held by local members that ‘knowledge’ resided only with the academic participants. From these entry points, the ECGs discussed past events, comparing and contrasting experiences, looked at reports, debated understandings and positions, and eventually identified the kinds of knowledge that needed to be produced. In both river basins, the framing that the ECGs developed tied knowledge production to predictive modeling and the data needed to sustain those models. Local participants as well as academics formulated what the models should do and were actively involved in setting them up, using them, and discussing the results. The models became powerful ‘objects’ in challenging what it was that both academics and locals thought that they knew about hydrological processes and management in both of the studied basins.

Finally, the objective of the ECGs was not to produce solutions. Rather, each ECG was able to create new knowledge about flood risk that was able to move the controversy on. Thus, one ECG produced an exhibition, attended by over 200 people, which in turn spurned a demonstration project by national government to take the work forward. This followed more conventional flood risk management procedures, ones which had previously rejected the kinds of ideas that the ECG produced. In the second ECG, the modeling suggested a series of small‐scale catchment interventions so as to reduce flood risk, which was adopted and is being delivered again through conventional flood risk management methods. In both cases, a second and critical result was the creation of new ‘social–hydrological’ publics, that is a grouping of ordinary people, communities, and academics, with a new sense of their social–hydrological knowledge and capable of intervening in ways that slowed down reasoning and, ultimately, created the space for new kinds of management.

Transdisciplinarity should not be equated with achieving consensus among participants. When dissenting voices are being suppressed then participatory processes can lead to a ‘depoliticization’ of water knowledge and management.^16^, ^47^ But when ways of acknowledging and working with conflict can be developed then this conflict may perform positive work by providing a motivation for disparate interests to engage in change, just like the agonistic–antagonistic mode of interdisciplinary research discussed above. The case of Environmental Competency Groups (ECGs; Box 4 and Table 1, projects P1 and P3) serves as an example of how agonistic transdisciplinary spaces may be created. In such fora, it is critical for participants to accept that they should be putting their own knowledge to the test. The notion of putting knowledge to the test is what can make transdisciplinary research inherently scientific, a point that is often missed when foregrounding fears of ‘watering down’ scientific knowledge with so‐called ‘lay opinions.’ All participants should put their knowledge to the test, and not simply those who are perceived to have a particular, ‘inferior,’ knowledge.^60^ One way of doing this is through deliberation. But, in the spirit of being scientific, it may be much more appropriate to work with material (e.g., maps and data) or virtual (e.g., models) objects that can challenge what we think we know. That is, knowledge begins to be produced when we reject what it is we think we know already and we do so through things and objects rather than discourse.

An equally important element is making something together, new knowledge. There is now some history of this being done in water research through participatory approaches to modeling (Box 3). Our experience suggests that participation makes the modeling process slower and more complex, and divergent with respect to existing modeling practices (Table 1).^29^ But it may make the modeling more salient, more reliable (through incorporating new knowledge) and better understood (by incorporating those who have to live with the model predictions). Participatory modeling may also play a heuristic role in enabling wider social learning,^61^ meaning new knowledge and practices that become situated within wider social communities beyond the level of the individual,^62^ which can move a problem forward.^60^

The focus upon modeling in transdisciplinary water research is not surprising given the dominance of modeling as a methodology. However, there are other ways in which knowledge is made through participation. This is notably the case in relation to ecological monitoring, such as for water quality to assess the status of fish populations.^63^, ^64^ It is often overlooked that the origins of ecological restoration, including river and catchment restoration, were not with government bodies, but with those who live with and use the river on a day‐to‐day basis. Gross^65^ argued that their participation became usurped by scientists, wishing to make restoration ecology a more formal science. It is perhaps ironic that this kind of water science has now seen a participatory turn, often under the label of ‘citizen science,’^66^ to recapture the role that the very making of knowledge plays in supporting sound water science.

## THE TENSION BETWEEN OPENING UP AND CLOSING DOWN MOMENTS IN TRANSDISCIPLINARY WATER RESEARCH

In this section we deepen the discussion of transdisciplinary water research by identifying important tensions that emerge in practice. Transdisciplinarity is inherently about opening up traditional modes of knowledge production; in terms of framing the research problem, the methodology and the knowledge that is considered permissible. This should open up the range of options for management intervention, too. While decisions on how to intervene will inevitably close down the issue periodically, the point here is to leave alternative routes of action open long enough, or reopen them again, so as to counter unsustainable and inequitable path‐dependencies and lock‐ins. The notion of opening up and closing down in policy appraisal has been theorized by Stirling.^50^ It has also been used by Westling et al.^67^ to reflect on their transdisciplinary project with a water utility that sought to develop a strategy for adaptation to climate change for the company. Following Westling et al., we will adopt the opening up/closing down motif in this section to reflect on the tensions we have experienced or expect in transdisciplinary research practice. Being reflexive is here itself a form of opening up our own positionality as researchers. Reflexivity has been identified as a critical element of transdisciplinarity.^6^, ^68^

The very idea of opening up traditional modes of knowledge production may be controversial among participants of transdisciplinary projects. Westling et al. identified a tension in their project between having to act authoritatively as scientists, so as to be recognized as experts by the water utility practitioners they were working with, and attempting to be transdisciplinary in recognition of the partiality of all expertise. Similar issues came up in projects P6 and P9 (Table 1) where some participants reacted negatively to our suggestion of opening up the process of hydrological modeling (‘You are the scientist—you tell us!’). In projects P1 and P3, the fact that the academic participants came from supposedly strong UK universities was seen as a political asset by local community members that would help their voice to be heard more readily with government organizations. It would be against the spirit of transdisciplinarity not to take such reactions seriously. The scope of opening up should be as much a matter of negotiation among the participants as anything in transdisciplinary research. If the group decides on a rather traditional division of labor then this is legitimate—as long as the opportunity for opening this up existed and will continue to do so should controversies arise (Box 3). As Jasanoff argued, it makes sense to view expertise as a form of delegated authority that can be revoked by democratic publics at any time.^69^ Realizing this may be a relief to those who believe transdisciplinary research will be ‘inefficient’ (not each and every aspect of research has to be opened up) but points to the need for flexibility on the part of the academics in responding to the ever‐changing demands of transdisciplinary projects.

Transdisciplinary research, through its explicit focus on socially relevant issues, is motivated strongly by intervention in the situation. Like any research, it is sensitive to how the research problem is framed; with great power in the hands of those who control the process, and constrained by those interventions that can be imagined at the beginning. Whoever initiates a transdisciplinary process will bring a preframing,^47^ already closing down the scope of knowledge that participants can produce. Explicit moves to opening up the problem definition again may conflict with the interests of those who initiated, fund or run the process, be they activists, Civil Society Organizations (CSOs), public bodies, private entities, or academics, as we experienced in projects P8 and P9 (Table 1). Academics, for one, may be limited by project deliverables (in case of research council funding, e.g.) in responding to any reframing of the research, as noted by Westling et al. for their project. In projects P1 and P3, we had to continually address the pressure imposed upon us by the funding body to produce ‘generic’ findings and, above all, techniques that could be transferred beyond the case studies chosen. Westling et al. also identified a tension between what matters to participants in transdisciplinary projects and what can be published in academic papers (which we are pushed to do). Renner et al.,^70^ in a comparison of five transdisciplinary water governance projects, also identified the divergence of academic and other participants’ expectations as a key challenge which needed careful management. In our experience, it is not that transdisciplinary research is academically uninteresting which prohibits publication. Rather, it is the time it takes to do transdisciplinary research that is ultimately taken away from writing papers, combined with the challenge placed by systems of academic evaluation (e.g., appointment processes and research assessments) where those who understand the value of transdisciplinary research may be poorly represented.

How well academics can respond to reframing the research will also depend on their own research culture and skills. In project P5 (Table 1), the choice not to use a model was impossible, despite participants questioning the usefulness of a model, partly due to project deliverables and partly because the team had already invested in the modeling process. Conversely, one can imagine a transdisciplinary project where the need for a model comes up but the researchers cannot respond as this would require a different make‐up of the team—a team that might be funded and assembled for this project only. Lack of skills was also the reason that in project P2 the team could not deal with a particular issue (sea level rise due to climate change) that was raised by participants. Where we had greater freedom to respond more flexibly to participants’ demands (project P8), and learned more skills to do so (in this case farm economic accounting), it might not have been the best science that got done just because we were a relative novice in this particular field. In projects P1 and P3, the work performed was crucially dependent upon a fixed‐term postdoctoral researcher's ability to translate ideas developed by the associated ECGs into working mathematical models, requiring working practices (e.g., the length of the working week) that many would find unacceptable. It appears that, in order to be able to respond to the dynamics inherent in transdisciplinary processes, we need a flexibility of using funds and bringing in skills that at universities is at odds with the largely disciplinary funding culture and the often short‐term, project‐based availability of researchers. In respect of funding, there are now examples of more flexible allocations of budgets that allow responding to research demands that emerge from transdisciplinary processes, not least providing financial assistance for nonacademic participants to conduct their own research.^71^ Regarding skills, consultancy‐oriented university outfits (e.g., www.csus.edu/ccp/) or nonprofit university spin‐offs (e.g., http://www.dialogik‐expert.de/en/) may be viable models for accessing skills as needed by transdisciplinary processes.

The scope of transdisciplinarity is further limited by what interventions in the situation can be imagined. In water research, this has a lot to do with institutions, technology and infrastructure. The ECGs described in Box 4 and associated with projects P1 and P3 (Table 1), e.g., formed explicitly out of dissatisfaction with the institutionalized procedures of managing flood risk which had effectively written the two localities studied off the map of flood risk intervention. Hydraulic infrastructure, like large dams, creates its own path‐dependencies because, once in place, it has to be maintained for some time and will have to feature, for better or worse, in future interventions. This path‐dependency is part of the reason why research has focussed on the supply side of water resources while side‐lining the demand side, a situation that has only recently begun to reverse.^51^ Hydraulic infrastructure has been part and parcel of the process of mutual legitimization of the hydrological cycle, hydrological expertise, water resources management, and centralized state control described by Linton.^20^

The question of framing immediately leads to the question of representation—who is and who else should be represented in a transdisciplinary process. Explicitly or inadvertently, the framing of problems and research questions will influence which knowledge holders are included or excluded.^72^ Here it is important to distinguish between transdisciplinary processes initiated by academics (somewhat artificial experiments) from those initiated by activists, CSOs, public bodies, or private entities (motivated by a problem). In experimental settings, academics may start with the idealistic objective that they can represent all those who are thought to have a stake in the problem. There are severe practical limitations in keeping group size manageable, balancing group composition, motivating and sustaining participants’ engagement, moderating the process, countering power imbalances, managing expectations, and preventing the process from being hijacked.^70^ But even if these could be managed, the framing of the problem and the definition of representation itself will always run the risk of excluding those with interests in other dimensions of the problem, even other problems.^4^, ^5^ In other situations, representation may be influenced, intentionally or otherwise, to fit a particular individual or institutional agenda. Among the nonacademic participants, too, there may be long‐established and hardened beliefs, both as to what the problem is and with regard to other participants. The scale at which the research is conceived to take place also influences who can or cannot contribute. 73

A key question is thus what motivates people to participate in knowledge coproduction. While participants may recognize their stake in a decision making or policy making process, they may not necessarily recognize their stake in a knowledge production process. This means that, especially in experimental settings, researchers need to think about, and make explicit, the benefits to participants (that may be tangible such as leading to further projects and plans that meet their interests, or intangible such as friends, networks, cooperation, or learning) as well as the costs (participants’ time and resources).^70^, ^74^ Situations initiated by activists, CSOs, public bodies, or private entities where academics come in may be more salient, but tend to come with a closed group of actors. From our experience (Table 1), there is a fundamental tension between salience and fairness, before the practical difficulties in balancing representation are even considered. As academics, we might thus give up completely on the traditional notion of fairness and decide to work explicitly with controversies (Box 4), and open up science‐policy processes that are perceived undemocratic in an attempt to counterbalance the prevailing discourses.

## CONCLUSION

A scientific decade on change in hydrology and society (IAHS ‘Panta Rhei’ 2013–2022) presents much needed opportunities for hydrology to engage with those disciplines that have traditionally been concerned with society, the social sciences, as well as with society itself. As our review has demonstrated, water knowledge is already produced widely within society, across certified disciplinary experts and noncertified expert stakeholders and citizens. Scrutinizing these knowledge practices and making them work together productively for a more complete understanding of human–water relations and the design of appropriate interventions is the aim of transdisciplinary research.

As our review made clear, much water research across and between traditional disciplines remains multidisciplinary, without integration of disciplinary paradigms. Understanding and management interventions may thus remain partial or even conflicting. The literature emphasizes philosophical differences between the generalizing and the interpretative traditions of science as well as methodological and communication differences as important barriers to integration. The social sciences typically take on a subordinate or service role in putatively interdisciplinary constellations. Out of the dissatisfaction with integration as synthesis that permeates the literature and our own experience we suggest a move toward more agonistic modes of interdisciplinary working where through critique, working together and different ways of seeing human–water relations we become simply ‘better researchers.’ This may instigate change also in our own disciplines, but not necessarily create any new ‘interdiscipline.’ In this endeavor, much inspiration can be taken, as we have done, from current thinking in sociology and anthropology.^98^, ^99^

There is much to be learned from those strands of social science that have examined both the social context as well as the consequences of particular scientific framings. Among those, STS have demonstrated how research practices embed and are embedded in particular social contexts, and who or what influences the production of any particular piece of knowledge. Political ecology, in turn, has scrutinized what this knowledge does in the world, with or for whom. Our review brought together cases of the cultural and political economy of water knowledge and uncertainty from these literatures. Hydrology and engineering have come under particular scrutiny. However, the social science studies of water, although often more reflexive, should be targeted symmetrically. Our review suggests that we need more empirical evidence on how exactly culture, politics, and economics have shaped water knowledge and in particular how and at what junctures this could have turned out differently.

While the coproduction of science and social order renders contingent the knowledge that is produced, acknowledging coproduction also provides opportunities for certified and noncertified experts to come together and coproduce water knowledge explicitly. Bringing perspectives, alternative knowledges, and implications into water politics where they were not previously considered counters potential lock‐in to particular water policies and technologies that may be inequitable, unsustainable or unacceptable. However, the literature suggests that, in order to allow academics to respond to the transdisciplinary demands more effectively, our science and funding institutions must begin to regard this type of research higher and allow for more time and continuity of science–society engagement. At the same time, the dynamic and somewhat unpredictable nature of transdisciplinary problems and research requires a flexible allocation of budgets and a flexible access to skills. Universities are particularly well placed here as they can draw from a wide range of disciplinary expertise. But the structure, funding, and staffing of departments must allow for both continuity as well as flexibility in assembling teams that fit the transdisciplinary problems as they unfold.

Recent transdisciplinary scholarship has foregrounded the productive use of conflict to challenge both certified (disciplinary) and noncertified knowledge to gain radically new insights into human–water relations, just like the agonistic‐antagonistic mode of interdisciplinarity. This mode of engagement directly challenges widespread instrumental notions of participation that have promoted consensus, thereby often maintaining the status quo both in terms of the prevailing knowledge as well as the existing distribution of power within a society. This is why transdisciplinary water research should engage explicitly with politics, but must remain attentive to closing down moments in the research process, such as framings, path‐dependencies, vested interests, researchers’ positionalities, power, and scale. Our reading of transdisciplinarity thus goes beyond applied research and expert consultation, both of which are sometimes subsumed under this label. At the same time, transdisciplinary research does not have to be overly constrained by the strongly normative tradition of the participation literature mandating democratically representative processes; an ideal that will never be fully achieved and would in any case conflict with problem closure. Where a political system is undemocratic or a democratic system fails, we suggest that transdisciplinary research may legitimately side with marginalized communities to produce knowledge that empowers these communities in the political arena.
